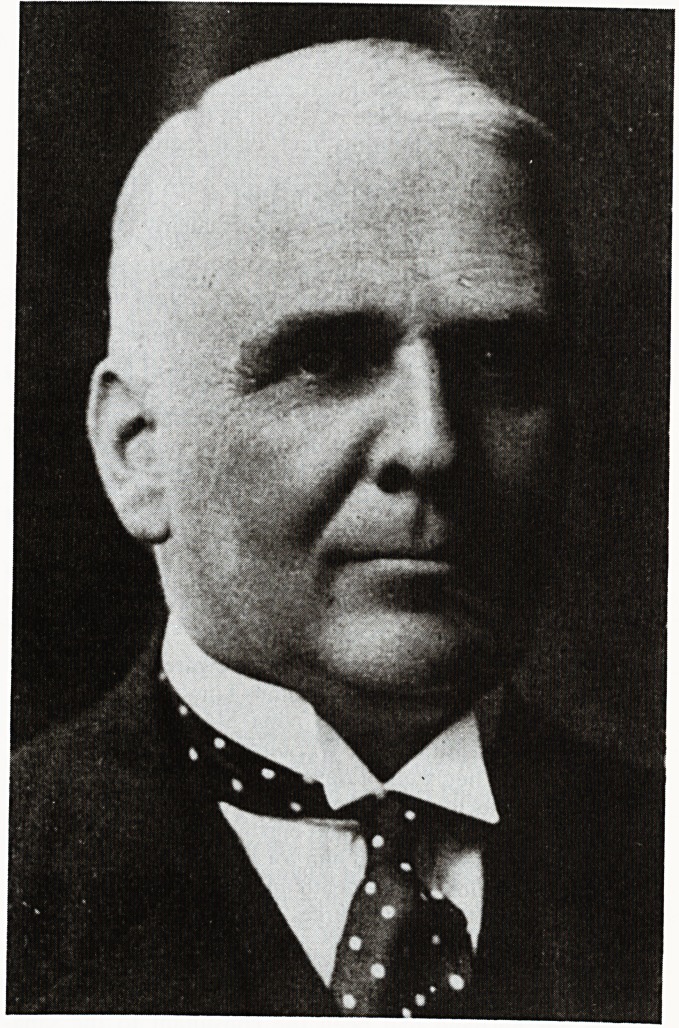# Carey Franklin Coombs 1879–1932

**Published:** 1989-11

**Authors:** Clive F M Weston

**Affiliations:** Department of Cardiology, University Hospital of Wales, Heath Park, Cardiff


### Carey Franklin Coombs 1879–1932

**Clive F M Weston**
Department of Cardiology, University Hospital of Wales, Heath Park, Cardiff

Carey Coombs was only 53 when he died in 1932. In the preceding 20 years he had won world-wide acclaim for his research and writings on rheumatic heart disease and had established himself amongst the best-known physicians in the provinces. In this he carried on a long tradition of famous Bristol medical men. He was universally liked and admired and would be more frequently quoted today had it not been for the virtual disappearance of rheumatic fever and cardio-vascular syphilis from all but the third world. Now he is remembered as an eponym for a murmur that few in this country will ever hear, yet a study of his life and work provides an insight into the development of early 20th century scientific inquiry where laboratory-based research was accomplished and applied for the benefit of society, rather than for the increase of knowledge alone. It also throws light upon a man who was highly respected and who should be recognised as a founding father of modern cardiology.

Carey Franklin Coombs was born at Castle Cary in Somerset on 5th September 1879, the son of Carey Pearce Coombs, the local general practitioner, and Mary Leslie Franklin of Coventry. Carey Coombs (senior) was a singular man—tall and ruddy, God-fearing and kind. His was a deeply religious household. The children, Carey Franklin included, attended the Congregational Chapel at Castle Cary where their father was treasurer. Though the family did not disdain people of other persuasions and were non-sectarian in their secular activities, his Free-Church upbringing was to be important when Carey Franklin entered the divided world of the Bristol medical establishment.

### Education and time in London

Carey Coombs (junior) was the only son. He had three elder sisters and one younger. He was sent to school at the charity school at Keyford on the southern outskirts of Frome. He later attended University College Bristol, gaining London matriculation in January 1896 and passes in chemistry and biology in University of London examination in 1897. With financial help from his father he enrolled as a medical student at St Mary's Hospital Medical School, starting his studies on 1st October 1987, being one of an intake of fifty-four. In the same year a certain Dr Frederick John Poynton was appointed Medical Registrar to the in-patients at the hospital. He was also a product of University College Bristol and St Mary's Hospital Medical School but was 10 years older than Coombs. His reputation as a teacher was exceptional and Coombs would have been among the many students who flocked to attend his rounds and his pathology demonstrations. Poynton was already interested in the aetiology of rheumatism and in 1900 published an article with Dr Alexander Paine expounding the theory of rheumatism as a result of streptococcal infection (1). He was to directly influence the young Coombs and became a life-long friend.

Coombs was a gifted and industrious student who lived up to his father's high standards. He passed the intermediate M.B. by July 1899, in the minimum possible time, and almost cleared the board of prizes in his final year.

In all he was awarded prizes in Medicine, Surgery, Pathology, Hygiene, Forensic Medicine and Psychological Medicine, and obtained a General Proficiency Scholarship worth £20 (2). He also won the Gold Medal in Clinical Medicine for an essay entitled "The clinical occurrence of excess of potassium idoxyl sulphate in urine". The St Mary's Gazette (edited by Poynton) carried the following remarks:

"His remarkable successes in the School during the last year are well known to all St Mary's men, and his future doings will be watched with interest." (3).

In 1903 he was elected Medical Registrar to the Hospital, moving out of the "House" and residing at No. 2 St Mary's Terrace, Paddington.

"A more popular appointment could hardly have been made", according to the Gazette, "our interviewer reports that he never had such a bashful patient as our new Medical Registrar; He had to chase his victim round three wards and a corridor before he was able to slake his fact-thirsty pen." (4). His duties as Registrar included the day-to-day care of the in-patients and the performance of autopsies on medical cases, for which he received £75 per annum. There was also a commitment to teaching students and the need to provide articles and case reports for the Gazette, though this latter task was performed "under some slight protest" (5).

Within a few days of becoming Registrar, Coombs was waylaid outside De Hirsh ward by Dr Poynton who invited him to develop an interest in rheumatic heart disease. For the twenty-three year old Coombs, this meeting with a man he so obviously admired presented an opportunity not to be missed. In a lecture prepared just before his death, and addressed to Poynton, Coombs states:

"That grain of mustard seed has given me a tree with many branches in which ideas of all kinds have found a lodging." (6).

Poynton advised Coombs to start by studying the case-notes of his father's old teacher Sir William Broadbent, so that he could gain insight into the many manifestations of rheumatic diseases. The inspection of Broadbent's notes led to the publication of an article in the Lancet entitled. "Some clinical aspects of the rheumatic infection" in 1904 (7). Here he described three types of reaction to rheumatic infection, namely transitory, protracted and malignant, and suggested that the organism responsible persisted in the infected tissue after all signs and symptoms had disappeared, and was capable of recrudescence. He looked upon endocarditis and mitral stenosis developing in adulthood as evidence of this recrudescence. These views were not particularly innovative, but they were a start.

During his student days at St Mary's, Coombs met a nurse called Nina May Matthews. She was also from Somerset where her father had been a farmer at Walton near Clevedon. Her family had broken-up following the death of her parents in the mid-1890's, and at the age of 19, she began nursing, first at Boston, Lincolnshire, and later Paddington. At the turn of the century she went to train in massage at the National Hospital (for Nervous Diseases) and in 1902 was appointed sister with responsibilities in the theatre. Here she worked with Sir Victor Horsley, a pioneer of neurosurgery who had performed the first successful removal of the spinal cord tumour with recovery of function. Through his fiancée, Coombs became a friend of Horsley, sharing as they did the same views on suffrage and temperance. As a wedding present Horsley gave the couple a silver cigarette box, which is still in the possession of the Coombs family.

Carey Franklin Coombs and Nina May Matthews were married at the parish church Wooburn, Buckingham on 5th October 1904. They were a devoted couple and throughout his life, Nina was to prove a great support for Coombs. She was a determined character who encouraged her husband in his work and almost idolised him for his successes. They had five children. Following his death and the resulting devastating reduction in financial resources, she cut expenses radically and took paying guests before moving to a smaller house. With the help of Coombs' former colleagues and the sympathy of both University of Bristol and Clifton College (where her husband had been the consultant to the sanatorium) she managed to ensure the completion of all her childrens' educations.

### Pre-War Bristol

1904 was an eventful year for Carey Coombs. At its beginning he was Registrar at a major London teaching hospital, he had just published his first article in a reputable journal and he had been successful in the M.D. examinations of the University of London. By its end he was a married man in general practice on Henleaze Road, Bristol. Quite why he left London is unclear. He certainly appeared well-placed to make a name for himself among the medical fraternity of the capital. Perhaps he never intended to stay there and looked upon his M.D. as the culmination of his academic work there. It is also possible that either he, or his wife, or both simply did not like life in the capital, for they had both been brought up in the country. Furthermore, if he wanted to be a General Practitioner why did he not go into practice with his father at Castle Cary, as was frequently the case in medical families?

The answer seems to be that Coombs entered general practice in order to gain experience, looking on it more as a necessary part of his training, as his father advocated, rather than an end in itself. Later in his life he explained his choice of Bristol in terms of its diversity and the resultant opportunities for epidemiological research. Therefore it can be concluded with some certainty that he never lost sight of an aim to combine clinical practice and research.

Carey Coombs spent less than 12 months in general practice and was appointed Registrar at the Bristol Children's Hospital within a year of leaving London, and in 1905 held this post simultaneously with that of Curator of the museum at the B.G.H. He continued to record cases of rheumatic heart disease and from these began to form his own opinions regarding the condition. He had notes on over 150 patients with valvular heart disease (not all rheumatic in origin), personally examined at St Mary's and 65 children with acute rheumatic fever seen as in-patients there and at the Children's Hospital. He also collected autopsy reports of children dying of heart disease in the Bristol area, and from these two sources attempted to describe more fully the natural history of rheumatic heart disease, especially in terms of physical signs. He was particularly impressed by the degree of myocardial involvement seen at one post-mortem of a child under his care at B.G.H. in 1906 (8), and this led him to embark on a painstaking study of the histological changes in both acute and chronic stages of the disorder. For this purpose he became the Demonstrator of Pathology at University College Bristol, where he worked under Professor Walker-Hall. In 1907, Coombs moved to the General Hospital as Assistant Physician.

The results of Coombs' early studies reinforced the views of Poynton; namely that rheumatic disease of the heart was a pan-carditis due to a blood-borne infection. It was shown that the major effects in the early stages were on the myocardium with consequent ventricular enlargement, and that only in later years did permanent valvular lesions become important.

Of 65 children with acute rheumatic fever he found a specific collection of signs in 38, among which were a blurring of the first heart sound, a mitral regurgitant murmur, doubling of the second sound, and a diastolic murmur (later known as the Carey Coombs murmur) (9). This diastolic noise was not a new finding. In fact Waler Cheadle had mentioned it specifically as a manifestation of the "rheumtaic state" in his Harveian Lecture of 1889 (10), believing it to indicate a narrowed mitral valve. Carey Coombs demonstrated the presence of the murmur in cases which, when examined after death, had no evidence of mitral stenosis. He explained the physical sign by postulating a relative stenosis of the valve, caused by a greater cubic expansion of the left ventricular cavity compared to the increase in cross-sectional area of the mitral valve ring (11). Likewise he thought the obvious mitral regurgitation resulted from stretching of the valve ring rather than involvement of the valve leaflets.

His histological work, largely funded by the British Medical Association, confirmed the findings of Aschoff (12). Coombs recorded the presence of submiliary nodules in all his cases of acute rheumatic carditis and carefully noted their positions within the heart and thier relationship to coronary artery branches. He concluded that cardiac rheumatism was due to a specific agent whose invasion of the heart via the coronary arteries led to nodule formation.

He also felt that invasion of the other organs could lead to the other manifestations of rheumatism, though his search for nodules elsewhere was not very successful. The identity of the infecting organism was a matter of debate, but Coombs was convinced by the work of Poynton and Paine that suggested the culprit was a streptococcus usually found in the gastro-intestinal tract. However he was never able to isolate the bacteria microscopically and mused that toxins produced outside the heart, perhaps in the tonsils, could be responsible for the myocardial damage.

Coombs' reputation continued to grow amongst Bristol medical circles. He was an active contributor to the meetings of the Bristol Medico-Chirurgical Society, in whose journal his work was frequently published. In November 1909 he formed a clinical society for the medical students and junior staff in order to bring forward "good cases in the wards". His lectures to the students were enjoyed and he was very popular with them. The following couplet appeared in the students' magazine, "The Stethoscope",

"C stands for Carey, that wonderful man,
Who cures broken hearts whenever he can."

He began to advocate the benefits of an amalgamation of the staffs and facilities of the Royal Infirmary and the General Hospital. Although he received much support from other members of staff, suspicions and prejudices thwarted all attempts at a union until after his death. At a dinner in 1909, marking the creation of the University of Bristol, he spoke of the great responsibilities placed upon clinical teachers, the need for coordination between the different institutions and less duplication of resources. Despite these views the two hospitals continued to develop separately. Coombs believed the opening of the new wing at the General Hospital in the week before the First War, together with the building of the King Edward VII wing at the Royal Infirmary, signalled the end of any hope of joining together.

**The War Years**

The outbreak of the war, whilst allowing the honorary staff of both hospitals to work with each other, did not come at a good time for Carey Coombs. His planned research was postponed. He was in his mid-thirties with a wife and three children to support (two more being born during the war years), and while he spent part of this time at home in Bristol he also served in Europe and the Middle East.

At the start of the war in 1914, Coombs was called-up and began work in the King Edward VII Memorial Building at the Royal Infirmary and at the new "Poor Law" hospital at Southmead, which together made up the "Second Southern General Hospital". Much of his work in the Southmead section was involved with assessment of men's fitness to return to fight. In particular he was interested in the contemporary debate regarding valvular disease of the heart, "V.D.H.", and disordered action of the heart, "D.A.H.", or irritable heart (13).

This latter condition, characterised by dyspnoea, palpitations and dizziness was thought by some to be a form of malingering, by others to be of psychological aetiology, and some to result from the action of bacterial toxins on the vagal innervation of the heart. Coombs, while advocating a "supra-cardiac" cause was sympathetic to such patients, writing:

"They will be more useful to their country making shells than being morally and physically knocked-down by them" (14).

He joined the British Expeditionary Force, attached to Number 32 General Hospital, during 1915, but was back in Bristol by the end of the year. In 1916 he was posted to Mesopotamia (present day Iraq), travelling via Marseilles, Egypt and Bombay, and arriving in Basra in the springtime. The hot weather was just beginning and to Coombs the country appeared "a vast colourless plain, almost devoid of trees, shimmering in the heat" (15). He journeyed 140 miles up the river Tigris to the 23rd Stationary Hospital at Amarah, where he found appalling conditions.

The hospitals were unsuitable. The difficulties in securing a site on high ground were insuperable, and tents were pitched on marshy land. There were also great problems with transportation of both men and supplies, so that up until May 1916, there were no electric fans or lights and no water purifying equipment. Coombs and a few friends boiled their water, but most men had to make do with gravity filters and bleach. Consequently there was a high incidence of illnesses, particularly diarrhoea, and it was calculated that the rate of admission to hospital due to sickness approached 1,500 per 1,000 troops each year. Coombs took great pains to record the large number of cases of depression and general exhaustion he saw and it is clear that he did not enjoy his time there nor the degree of suffering he witnessed around him.

One of the few advantages was the chance to renew his acquaintance with Sir Victor Horsley, now a colonel, who had arrived in Basra in March 1916. Unfortunately Coombs was becoming unwell himself, suffering weight-loss, diarrhoea (initially thought to be cholera), and finally a type of nephritis. He saw a great deal of Horsley during his last weeks at Amarah, but eventually left there on 8th June 1916 and arrived back in Southampton on 23rd July. Any elation he may have experienced on his return was tempered by the first newspaper he read, which reported the death of Horsley from sun-stroke at Amarah one week before.

Carey Coombs convalesced speedily in England and was transferred to active service in 1917, when with promotion to the rank of Major, he was put in charge of the medical wards of a base hospital in the Rouen area of northern France. Coombs was sent to collect information on chemical injuries sustained by soldiers who had been gassed during the battle of Ypres in July, though this data was not made public until after the war (16).

He also, with more than a passing self-interest, studied cases of nephritis among the troops, amassing notes on 160 cases in his seven month stay in Franch (17).

In the same year he was elected a Fellow of the Royal College of Physicians of London. He was restored to the medical establishment on 26th January 1918 and remained in Bristol for the rest of the war.

**Post-War Researches**

At the war's end, Coombs took up his research into rheumatic heart disease again. With a grant from the Colston Research Fund and the help of Dr D S Davies, the Medical Officer of Health for Bristol, he surveyed the death registers for the city during the years 1876 to 1913. He discovered that the mortality due to rheumatic heart disease had fallen over the 37 years, but not as sharply as the death rate generally (18). Using the "spot map" method pioneered by William Budd, a former Bristol physician, he failed to show any relationship between the incidence of the disease and geology, housing or proximity to rivers, all factors previously considered important. He did demonstrate an inverse association between incidence and annual rainfall. Coombs also noticed that when the city boundaries had been extended to take in a large area of countryside, there had been no increase in mortality. Not only did this sow the seeds for a bigger study involving the counties surrounding Bristol, but it also convinced Coombs of the importance of fresh air in the treatment and prevention of acute rheumatism, as was already the case for tuberculosis patients.

Coombs was very much aware of the loss of continuity that the war had caused, especially with respect to his case-notes. However he was too busy to chase-up the "lost" patients himself. In 1921 he successfully applied for a grant from the Medical Research Council, with which he employed a young man called Emery. Emery's job was to visit the last known addresses of Coombs' patients, of which there were about 700, and encourage them to attend the General Hospital for a reexamination by Coombs or his colleague from the Royal Infirmary, Dr Charles E K Herapath. If the patient no longer lived at the address, Emery had to track them down. The vast majority of patients were found in this way, enabling Coombs to study the natural history of rheumatic heart disease over a period of 15 years.

In 1920, the Ministry of Pensions appointed consultants in Cardiology to assist in the treatment and assessment of cardiac patients. Carey Coombs took on this responsibility for the south-west region. These consultants met occasionally in London to discuss Ministry business, but also took the opportunity to talk about interesting cardiological topics. These meetings were enjoyed by all who attended, and formed the impetus to establish the Cardiac Club, the forerunner of the British Cardiac Society (19). The official accoucheurs were Coombs, Tom Cotton, John Cowan of Glasgow and William Hume from Newcastle.

Coombs had the honour of chairing the third Annual meeting which took place in Bristol on 5th June 1924, at which he introduced the subject of "Heart disease in children", and secured the election of his friend Herapath into the Club.

Another important event that took place in 1924 was the completion of Coombs' book, "Rheumatic Heart Disease", published by John Wright & Sons of Bristol at a price of twelve shillings and sixpence (20). Its 376 pages contained the sum of Coombs' experience and thoughts on the subject, gained in more than twenty years research, and covered aspects of histology, history and clinical manifestations, treatment and prevention. The introduction was written by Poynton who had started the young Coombs on this road in the first place and with whose theory of the aetiology of rheumatism the latter readily agreed.

The style of writing, as in his previous publications, was clear and concise, with specific cases used to illustrate the important points. The book was therefore easy to read and was greeted by the Lancet as a "valuable monograph". In particular, the chapters on treatment (rest, salicylates and hospital schools) and prevention (education and prospective studies) were of great interest. From this time he began to diversify his interests and to become increasingly occupied with committee work.

His authority in the field of rheumatic heart disease was recognised when, in the same year, he was appointed to a special sub-committee of the British Medical Association's Science Committee, whose brief was to inquire into the prevention, detection and treatment of cardiac disease in children. Both Coombs and Poynton were among the six members of the group who, with the help of Sir Thomas Lewis, reported on the subject in July 1926 (21). He strongly supported the proposals regarding the provision of accommodation for institutional care of rheumatic children, where they could receive controlled rest, with plentiful sunlight and fresh air, concurrently with education over a period of months. In March 1927 he delivered the Chadwick Lectures on the causes and prevention of rheumatism (22), in which he emphasised the part played by such accommodation by quoting Longfellow's couplet:

> "Joy and temperance and repose
> Slam the door in the doctor's nose".

The post-war years saw Coombs' influence growing in Bristol as well. He had moved into a house in the affluent suburb of Clifton, at 3 Pembroke Road. No fewer than 12 doctors lived in that street alone, including two senior physicians from the General Hospital. He was held in high esteem by his peers, and following a postal vote of all the practitioners in the city, was elected Honorary Secretary of the Bristol Medical Committee, the first local advisory council in the country (23). He also became joint editor of the Bristol based "Medical Annual" sharing this responsibility with Mr Rendle Short (later Professor of Surgery at the Royal Infirmary) with whom he formed a firm friendship.

Coombs was invited to give the Long Fox Memorial Lecture to the University of Bristol on the causes of heart disease.

In 1927, upon the resignation of Dr J Odery Symes, Carey Coombs became a full physician at the General Hospital.

Coombs' plans for research had not ended with the publication of his book. He longed to set up a post-graduate research unit along the lines of James Mackenzie's at the London Hospital and Lewis' at University College Hospital. He therefore began seeking financial support. In 1927 the "Centre for Cardiac Research" was officially opened by Thomas Horder, a fellow member of the Cardiac Club. The Centre was housed in cubicles, formerly nurses' bedrooms, on the first floor of the Octagon tower of the General Hospital, this accommodation being loaned by the management of the Hospital for an initial period of three years. The main funding came from the R L St J Harmsworth Memorial Fund (24), but there were also contributions from the Medical Research Council and the Colston Research Society. A Cambridge string galvanometer electrocardiograph was provided by the University, on loan from the Physiology Department, and because of this the Centre was renamed the "University Centre for Cardiac Research".

The grant from the Colston Society provided £200 per year research fellowship for an assistant for Coombs. The holder of this fellowship was the then Medical Registrar, Dr C Bruce Perry (later Professor of Medicine at the Royal Infirmary). As part of his training for the post, Bruce Perry was sent to be Poynton's House physician at Great Ormond Street, spending two afternoons each week at the London Hospital with Evan Bedford and John Parkinson, learning how to take an ECG. These were the days when obtaining an ECG was a laborious business, involving taking a trace, developing the plate and making the print. Over 2,500 such recordings were made during the first three years of the Centre alone. The majority of these were on hospital patients, but a service was also provided to private patients, who would contribute a small sum towards the upkeep of the unit. Money was also received from the Bristol Education Committee because the Centre provided a referral service for children who were suspected of having cardiac disease.

In 1923 Coombs and Herapath began receiving, from the schools' medical officer, Dr Askins, such children who had been examined and found to have abnormal symptoms or signs. Three years later, following the publication of the B.M.A. report into rheumatism, the clinic was extended to take children from the surrounding counties. Coombs circulated an information package to the schools describing the early signs of rheumatic fever: an early example of health education. He persuaded the Medical Officers of Health in these areas to make the disease notifiable, and organise for all cases to be seen by a selected physician.

These children, together with 337 cases sent by private practitioners, accounted for the 754 cases analysed at the Centre of Cardiac Research and reported at the annual meeting of the Royal Society of Medicine's section for the study of disease in children in Bath 1931 (25). This demonstrated conclusively that the incidence of the disease per 1,000 of the population was five times higher in Bristol than in the neighbouring counties. The collaboration of these provincial physicians continued until Coombs' death, and formed the framework for the West Country Physicians Club, which Perry organised some years later.

Carey Coombs' beliefs regarding the management of children with rheumatic fever have been alluded to earlier. There was already an orthopaedic hospital of 36 beds on Grove Road in Redland, Bristol, which predominantly took in children with osteomyelitis and tuberculous spinal disorders. With the help of another colleague, Professor Hey Groves (the profesor of surgery), Coombs began campaigning for a residential hospital to be built outside Bristol. At first there was a lot of opposition to this from those who foresaw the difficulties of transporting the parents from central Bristol at visiting times, and the response to the appeal was disappointing. Coombs felt this apparent lack of care very keenly, and expended vast amounts of time and energy in getting the scheme off the ground. Finally, with the amalgamation of the

Redland Orthopaedic Hospital and the Bristol Crippled Childrens' Society, the new Winford Orthopaedic Hospital was built on the foothills of the Mendips, south of Bristol. Even after the opening by Prince George on 31st May 1930, the hospital remained short of funds, much to Coombs' chagrin (26).

Though primarily an orthopaedic hospital, there were twenty beds allocated for cardiac cases and this number soon increased. Perry and Herapath were in direct charge of the patients with Coombs and Poynton being Honorary Consulting Physicians. The Winford project was close to Coombs' heart, and the family connection continued long after his death with his sisters being annual subscribers to the hospital and his wife serving on the Management Committee from 1935 until 1948.

### Cardiovascular Syphilis

Coombs had first become interested in cardiovascular syphilis as a student at St Mary's. He remained cognisant of the advances in this field, particularly regarding the treatment of the condition, though he was too busy with other matters to contribute directly. After the war he was mindful of the great increase in the incidence of primary syphilis consequent upon it, an aspect of the war.

> "as sordid as it has been constant in
> all modern campaigns" (27)

and sought to estimate the repercussions to health that this would have in years to come. His work on the topic took very much the same form as that on rheumatic heart disease, namely a combination of clinical and histological reports.

In order to furnish a basis for a review, he analysed the records of 103 patients with cardiovascular syphilis seen by himself in all parts of his practice in the twelve years since the war. He also enlisted the help of Dr A L Taylor, then the pathologist at the General Hospital, to examine the post-mortem records of 1,750 patients dying at the B.G.H. from 1919 until 1929. His pathological enquires took the form of microscopy of sections obtained from 21 hearts of affected patients. Once again this was laborious work. Around 120 sections were examined from each heart. However, whereas in his previous studies on rheumatism Coombs had to prepare all the specimens himself, he now had the assistance of technicians. The fruits of this labour on syphilis were presented to the Royal College of Physicians when Coombs delivered three Lumleian Lectures in 1930 (28). In holding this lectureship he followed in the footsteps of Harvery, Osler and Horder, an honour which he recognised with pride.

The post-mortem study revealved that syphilis accounted for 12.7% of all cardiovascular deaths in the ten year period, which was almost twice as much as his clinical records suggested. This discrepancy was explained by the inclusion of sudden deaths, mistaken diagnoses and subclinical infections. The main finding of the histological study was an aortitis and a reduction in the elasticity of the aorta, even in the absence of an aneurysm. The aortitis was felt to be a consequence of inflammatory infiltration from the peri-aortic lymphatics, and resulted in a tethering of the aortic valve cusps leading to aortic regurgitation. Another striking observation was the lack of any specific myocardial change.

The clinical review revealed the early symptoms to be dyspnoea and cardiac pain, with an average lifespan of 2.5 years from the onset of these complaints. In over half his patients there was evidence of syphilitic aortic regurgitation. Coombs differentiated the murmur of syphilitic aortic regurgitation from that of rheumatic aetiology of the position of maximal loudness: the former being heard best at the right sternal edge and the latter at the left. He also mentioned the practice of Herapath to use a wooden stethoscope to listen for these murmurs.

He was also struck by the frequency with which syphilis caused chest pains compared to the rare association of the disease with myocardial infarction. Bruce Perry undertook to inject the coronary arteries of five "syphilitic" hearts with radio-opaque bismuth, and using X-rays demonstrated the absence of any stenoses along the length of the arteries, while their origins were critically narrowed. Coombs explained the chest pains in terms of poor diastolic coronary blood flow, and the infrequency of infarction because of the lack of atheroma and consequent improbability of coronary thrombosis.

His theory of the aetiology of cardiac pain was based on the ischaemic model, namely that a failure of supply of oxygenated blood to the myocardial cells resulted in pain, and he used as evidence for this belief the similar pains experienced in syphilis, coronary disease and pernicious anaemia. This is not to say that Coombs attached no importance whatsoever to the aorta, and believed that the loss of elastic recoil of the atheromatous aorta could also be implicated as a mechanism for poor diastolic coronary flow.

### Coronary Artery Disease

The subjects of coronary thrombosis and myocardial infarction occupied Coombs towards the end of his life. These were newly-recognised disorders, the first diagnosis of myocardial infarction made during life being generally credited to Obrastzow and Straschesko (29), who published their cases in the German literature in 1910. However, in the early 1900's Coombs attended a clergyman who had high blood pressure and had suffered a stroke. In his presence the patient had an attack of severe sub-sternal pain, and soon after developed acute pericarditis. Coombs postulated that what had happened in the man's cerebral circulation had now occurred in his coronary circulation, namely a coronary thrombosis. Fortunately for the clergyman, though not for Coombs, the attack was not fatal, and Coombs was robbed of the chance to confirm his diagnosis by post-mortem, and with it a more significant place in history (30).

The 1925 meeting of the Cardiac Club devoted a large proportion of the proceedings to a discussion of "Anaemic necrosis of the heart", introduced by the then Secretary, Dr A G Gibson, but including contributions from most of the members. This was very much a "state of the art" symposium, and similar meetings were held under the auspices of both the Royal Society of Medicine and the British Medical Association over the next few years. Carey Coombs and Geoffrey Hadfield, the pathologist at the Bristol General Hospital, presented their data regarding the consequences of coronary obstruction: acute ventricular failure, chronic cardiac failure and sudden death before diagnosis. They thought that infection could be a precipitating factor, because of the raised white blood cell count and pyrexia that often accompanied the attacks.

By 1927 Coombs had collected 36 cases of coronary thrombosis, though surprisingly no post-mortem examinations were performed on the 18 patients who had died, and by 1932 he had details of 144 (31). From this latter group he was able to obtain information pertaining to the prognosis in myocardial infarction. He found that about one third died shortly after the attack, a further third died within a year of the attack and that the remainder survived with variable disability. Although there were obvious predisposing factors: age, sex, high blood pressure and a family history of similar disease, the prognosis was dependant on the severity of the attack rather than the background to it.

The attack was judged severe in the presence of a pericardial rub, persistent tachycardia, a fall in systolic blood pressure and a reduction in pulse pressure.

This work on coronary artery disease was published posthumously. In late August 1932 Coombs took a rare break from his various medical and administrative duties and went on a family holiday to Scotland. While walking up a particularly steep slope in the Cairngorms with his youngest son, Richard, he suddenly stopped, and after a brief rest carried on gently by a less arduous route. He had angina. The significance of this was not lost on Coombs.

Within a few weeks he was admitted to his own ward in the Bristol General Hospital, having collapsed at the bottom of Park Street while walking with Dr R H Perry, the Medical Officer of the city. Although he did not suffer pain on this occasion, it was felt that he had undergone a "coronary accident", and was extremely ill. He rallied, and after a number of days was well enough to continue work, in a fashion, from his hospital bed. He was allowed visitors, and many of his colleagues came to see him. As weeks went by his strength returned and his confidence obviously grew. He enthused with Poynton about a lecture he was due to deliver at University College Hospital concerning his latest experiences in rheumatic heart disease, and joked that his enforced bed-rest made him feel like the "small boy whose nether garments had been hidden". His secretary, Miss Michel Clarke, attended him, and with her help he corrected various manuscripts.

Ironically, one such amendment was a letter by Coombs to the Editor of the Bristol Medico-Chirurgical Journal (who received it the day before Coombs' death) relating to his paper on the prognosis in coronary thrombosis. In an extra paragraph Coombs stressed that survival for more than one year following a myocardial infarction was often associated with complete recovery, including a return of full-time work. He explained the addition by saying,

> "I think it is only fair to others in the same boat to dwell a little more than I have done on the bright side.".

Unfortunately, in spite of these brave and optimistic sentiments Carey Coombs died suddenly on 19th December 1932 (32).

As mentioned earlier, Coombs left a young family not too well off. His eldest son, Noel, was already a qualified doctor, and the next, Franklin, was at Bristol Medical School. His only daughter Mary, was a trainee nurse. The two remaining sons were still in full-time education, Martin (later killed while serving in the R.A.F.) was doing an engineering course at Cambridge and had to return to complete his studies in Bristol, and Richard who was a day-boy at Clifton College and later got a degree in geography at Bristol University.

Coombs' colleagues arranged a collection, the interest from which was paid to his widow until the children were educated.

When this was accomplished the money was utilised to provide a prize, and the Carey Coombs Memorial Lecture, the first such being delivered by Lawrence O'Shaughnessy on the subject of "Cardio-omentopexy", in 1937.

Coombs' memory lives on in Bristol in other ways, largely due to the influence of Bruce Perry, his former research registrar, who became Professor of Medicine in 1935. When the General Hospital and the Royal Infirmary finally amalgamated in June 1940, Perry named one of the medical wards "Carey Coombs Ward" after his old chief, though as there were by that time no medical beds at the General Hospital this ward was located in the old building of the Infirmary. There was also a "Carey Coombs Research Fellowship", started in 1952 and continuing until today, the original holder being Dr D W Barritt.

### Coombs: The Man

It is clear that, particularly towards the end of his life, he was overwhelmingly busy with research and committee work. He was renowned as a good and enthusiastic teacher of students, junior staff and his colleagues. His penultimate houseman recalls occasions when Coombs was called back to the General Hospital in the evenings to see urgent cases. If the case was of interest, the junior doctor would return to his room to find a full written case-history, together with text books open at the appropriate pages, lying on the table. Similarly, if in the course of a ward round Coombs found any clinical observation of importance he would make sure that everyone had seen it and understood its relevance before he passed on to the next patient.

He took such duties extemely seriously and prided himself on the amount of work he got through. He spent a lot of time examining, both at Bristol and elsewhere, and was thought, if anything, to be too kind, especially to the female students. His complete charm and courtesy tended to suggest that he accepted everything that the candidate said, whereas he actually expected the basic facts to be known exactly. Many examinees were shocked to discover they had failed and probably blamed the other examiner for it was inconceivable that it was the "nice one's" fault.

He was most particular on politeness and punctuality, values that he himself exhibited and expected in others. He would quote the motto "Manners maketh man", and looked upon rudeness as a greater sin than ignorance.

It irritated him if he was late for any appointment or kept a patient or another doctor waiting. Similarly, if any of his children were late coming down for a meal they would receive very dark looks, though no actual punishment. Coombs maintained discipline through respect.

Relaxation during the working week was difficult to achieve and was mostly in the form of reading: medical books, religious works and also articles on history, archeology and architecture. When his father was alive Coombs would drive the family down to Castle Cary for a weekend in the country, and later, family holidays were spent on farms all over Great Britain. Though they lived in the city, all the Coombs' children developed a strong love of nature, encouraged more actively by their mother. Coombs adored hill-walking and would sometimes cover twenty miles in a day. He was not over-keen on driving which was a very necessary part of his job as a visiting consultant, and enjoyed the freedom that these breaks provided, often travelling to the base destination by train.

There was an ambience of the medical world within the house in Bristol, not only because of his intense preoccupation with medicine and his wife's admiration of him and his work, but also because of the developing interest in medicine exhibited by his two eldest sons. There were also a stream of visitors from other hospitals and institutions, both in this country and abroad, who experienced Coombs' friendly hospitality. However there was always encouragement for the children to pursue other interests, whether it took the form of, for example, Franklin's love of ornithology, Martin's interest in motorbikes or Richard's in geography and history.

It was a happy household, if not, by present-day standards, a little austere. Coombs would take a cold bath in the mornings and expected his children to do likewise. Although not rigidly tee-total, he did not drink alcohol in the house, and what few meals he ate away from home were either medical dinners or taken on journeys. There were always traditional family prayers before breakfast, as there had been at his father's house in Castle Cary, and he would occasionally read passages from the Bible aloud at mealtimes. The whole family would attend the Congregational Highbury Chapel in the suburb of Cotham, each Sunday, usually walking the mile or so from home to church, though Coombs frequently drove down to the General Hospital beforehand.

It should not be thought that he was dull or morose. In fact he was notorious for his cheerfulness and supply of amusing stories. He was a genial companion who radiated sincerity and openness. Handsome and well attired, he had a style and clarity of speech that was both informative and unpretentious, and although there was a private side to him, when he died there were a large number of people who felt a personal loss.

John Poynton wrote, "With the untimely death of Dr Carey Coombs passes a friend bound to me by the ties of affection, regard and comradeship. . . . Such a man makes me proud to be a doctor.".

Rendle Short commented, "Many consulting physicians are highly respected by their professional brethren for their works sake; some are loved for their own sake. It is no funeral flattery, but the barest truth, to say that Carey Coombs enjoyed in full measure the double tribute .. . . But there was something more, and we loved him. . . . Usually a death in medical circles is like a stone dropped on water; there is a splash, some spreading circles or wavelets, then all is still again. Not so in this case. (33)

It is now over fifty years since the death of Carey Coombs. Though Coombs would have wished to have lived to see the final amalgamation of the Bristol hospitals and to witness Winford Hospital put on firmer financial foundations, he could be proud of many things. The major achievements being his help in the founding of the Cardic Club, the opening of the Centre for Cardiac Research, the building of the hospital-school at Winford, and the coordination of a large number of medical practitioners in his studies of rheumatic heart disease.

It cannot be said that Coombs' work displayed great genius, although his book and his Lumleian lectures are both landmarks in their respective fields. His researches were built, not on sudden inspiration, but upon meticulous thought and hard work. He was a superb organiser whose enthusiasm motivated those around him. He was primarily a physician whose upbringing through Victorian values allowed him to span the gulf between the art and science of medicine.

### Acknowledgements
I should like to thank the following people for their help: Dr. Noel Coombs, Dr. Franklin Coombs, Mr. Richard Coombs, Prof. C. B. Perry, Dr. Douglas Johnson, Miss B. A. Michell Clarke, Dr. Juliet Rogers, Dr. C. Rendle Short, Dr. Arthur Hollman.

### REFERENCES

1. POYNTON, F. J. and PAINE, A. E. (1900) "The etiology of rheumatic fever", *Lancet*, ii, 861-9. For more details of Poynton's career see Munk's Roll, vol IV p 454–4.
2. These awards are listed in Coombs' student record card, together with details of clinical attachments from January 1900 until May 1901.
3. St Mary's Hospital Gazette, 1901, VII:154.
4. St Mary's Hospital Gazette, 1903, IX:54.
5. St Mary's Hospital Gazette, 1903, IX:87.
6. This quotation and the account of the meeting between Coombs and Poynton is found in "Thirty years progress in the study of rheumatic heart disease", *Bris Med-Chir J.*, 1933, **50**, 93–112, which Dr Bruce Perry delivered to the University of London soon after Coombs' death.
7. COOMBS, CAREY F. (1904) "Some clinical aspects of the rheumatic infection", *Lancet*, i, 565–8.
8. COOMBS, CAREY F. (1922) "Streptococcal infection of the heart", *Quart J Med*, **25**, 114.
9. In "Rheumatic carditis in childhood" *Bris Med-Chir J*, (1907), **25**, 193–200. Coombs states: "Next the apical second sound becomes doubled and then, but often not till some time after, a murmur is added to the second half of this second sound. This, hard to distinguish at first, lengthens and strengthens till at last it runs into the beginning of the next cycle, becoming, in fact, a pre-systolic murmur. This is not as rough and loud as that of mitral obstruction and it is not due to valvular disease.".
10. CHEADLE, W. B. (1889) "Various manifestations of the rheumatic state as exemplified in childhood and early life", *Lancet*, i, 821–7.
11. In "Rheumatic myocarditis" *Q J Med*, (1908), **2**, 26–47. Coombs explains that " . . . a relative stenosis is established, and blood sucked through this relatively small mitral ring by the rapid expansion of an enlarged ventircle is thrown into vibrations which are appreciated by the clincial observer as a mid-diastolic murmur.".
12. Although Aschoff described the submiliary nodules a few months before Coombs found them, Coombs claimed priority in realising their aetiological importance when he delivered the Long Fox Memorial Lecture in Bristol on 9th December 1925.
13. For a discussion of this topic and its relevance to the later formation of the Cardicac Club see Joel D. Howell, "Soldiers heart: The redefinition of heart disease and speciality formation in early twentieth century Great Britain" *Medical History*, (1985), suppl no 5, p. 34–52.
14. COOMBS, CAREY F. (1915) "Cardiac diseases and disorders in warfare", *Bris Med-Chir J.* **33**, 149–56.
15. COOMBS, CAREY F. (1916) "Medicine and surgery in Mesopotamia", *Bris Med-Chir J.*, **34**, 136–44.
16. COOMBS, CAREY F, STACK, H. E. and ROLFE, R. (1920) "Poisoning by mustard gas", *Bris Med-Chir J.*, **37**, 151–62.
17. COOMBS, CAREY F. (1918) "Army nephritis", *Lancet*, i, 495.
18. COOMBS, CAREY F. (1920) "The incidence of fatal rheumatic heart disease in Bristol: 1876–1913.", *Lancet.*, ii, 226.
19. COWAN, JOHN et. al., (1939) "Some notes on the Cardiac Club", *Brit Heart J.*, **1**, 97–103, and CAMPBELL, MAURICE. (1962) "The British Cardiac Society and the Cardiac Club: 1926–1961.", *Brit Heart J.*, **24**, 673–95.
20. COOMBS, CAREY, F. (1924) "Rheumatic Heart Disease.", Bristol, John Wright & Sons Ltd.
21. *Brit Med J.*, (1926) suppl, 9–25.
22. COOMBS, CAREY, F. (1927) "Rheumatic infections of childhood.". *Lancet*, i, 579–80, 634–5, and "The chronic rheumatic diseases", *Lancet*, i, 739–41, 802–3.
23. The medical advisory committee was made up of representatives from members of the following groups: Bristol medical panel, general practitioners not on the panel, women practitioners, whole-time administrative officers, and hospital staff. Their role was to advise public bodies as to the views of the local medical profession and to forge greater links among their number.
24. The fund was set up by the Harmsworth family to promote research into endocarditis. Three members of the family had died of this disease. When a cure for the disease was found the capital was to be given to the successful researcher. In fact, when it became clear that Penicillin was an effective treatment, the money was divided between Flemming, Florey and Chain. When Coombs died the funding of the Centre for Cardiac Research finished, although Bruce Perry was commissioned to write a book on bacterial endocarditis. The M.R.C. grant was mainly for clerical expenses in the running of epidemiological studies. The work of the Centre is described by Coombs in "The work of the University Centre of Cardiac Research: 1927–31." *Bris Med-Chir J.*, (1931), **48**, 179–88.
25. COOMBS, CAREY, F. (1931) "The incidence of juvenile cardiac rheumatism in the west of England.", *Proc Roy Soc Med.*, **24**, 1611–2.
26. The initial cost for the Winford project was £40,000 but by the opening this had risen to £64,000 and there was an outstanding debt of £18,000.
27. COOMBS, CAREY, F. (1932) "The diagnosis and treatment of syphilis of the aorta and heart.", *Q J Med*, **1**, 179–211.
28. The Lumleian Lectures were published as "Syphilis of the heart and great vessels.", *Lancet*, (1931), ii, 227–31, 281–6, 333–9.
29. OBRASTZOW, W. P., STRASCHESKO, N. D., (1910) "Zur kenntnis der thrombose der koronararterian des herzens.", *Zeits Klin Med*, **71**, 116–32.
30. COOMBS, CAREY, F. (1927) "Coronary obstruction.", *Bris Med-Chir J.*, **44**, 249–56.
31. COOMBS, CAREY, F. (1932) "Prognosis in coronary thrombosis.", *Bris Med-Chir J.*, **49**, 277–84.
32. Although the obituary reports state that he died of coronary thrombosis, it is odd that both the initial attack and the fatal episode were painless. In fact at later examination there was no obvious cardiac infarction and the coronary arteries were good.
33. These and other memorials are found in *Brit Med J.*, ii, 1267, 1171, and *Lancet*, (1932), ii, 1361–2, and Medical Annual.

## Figures and Tables

**Figure f1:**